# Accelerating Catalyst Materials Discovery With Large Artificial Intelligence Models

**DOI:** 10.1002/anie.202526150

**Published:** 2026-02-17

**Authors:** Di Zhang, Yuanzheng Chen, Chuanyu Liu, Yan Liu, Hongliang Xin, Jiayu Peng, Pengfei Ou, Hao Li

**Affiliations:** ^1^ Advanced Institute for Materials Research (WPI‐AIMR) Tohoku University Sendai Japan; ^2^ School of Physical Science and Technology Southwest Jiaotong University Chengdu Sichuan China; ^3^ Department of Materials Design and Innovation University at Buffalo Buffalo New York USA; ^4^ Department of Chemistry National University of Singapore Singapore Singapore; ^5^ Department of Chemical Engineering Virginia Polytechnic Institute and State University Blacksburg Virginia USA

**Keywords:** AI for catalysis, artificial intelligence (AI), data‐science, large language models, machine learning interatomic potentials

## Abstract

The integration of artificial intelligence (AI) into catalysis is fundamentally reshaping the research paradigm of catalyst discovery. Unlike traditional trial‐and‐error approaches, AI‐empowered data‐driven technologies, particularly large AI models such as universal machine learning interatomic potentials (MLIPs) and large language models (LLMs), offer unprecedented capabilities in exploring complex spaces, predicting catalytic performance, and accelerating rational design. Standing at the forefront of data‐driven science, we underscore how databases, universal MLIPs, and LLMs are revolutionizing the traditional catalysis paradigm and bridging the ontology‐concept‐computation‐experiment continuum. We then demonstrate significant recent progress, and discuss their potential and challenges in the catalytic field. By leveraging cutting‐edge universal MLIPs and LLMs, researchers can conduct large‐scale simulations, highly efficient data acquisition, training, and prediction, and even self‐directed research in the field of catalysis. Looking ahead, these advantages enable the rapid development of target catalysts, which will be propelled by integrated universal MLIPs, multimodal LLMs, and automation systems. Developments in these domains will pave the way toward AI‐empowered closed‐loop platforms and cross‐disciplinary Digital Materials Ecosystems that broaden the discovery landscape and foster cross‐materials innovation, marking the dawn of a new era in which catalyst materials discovery is perpetually accelerating.

## Graphical abstract

AI‐empowered catalysis research via integrated database platform, universal machine learning interatomic potentials (MLIPs), and large language models (LLMs).

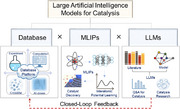

## 1 Introduction

**FIGURE 1:**
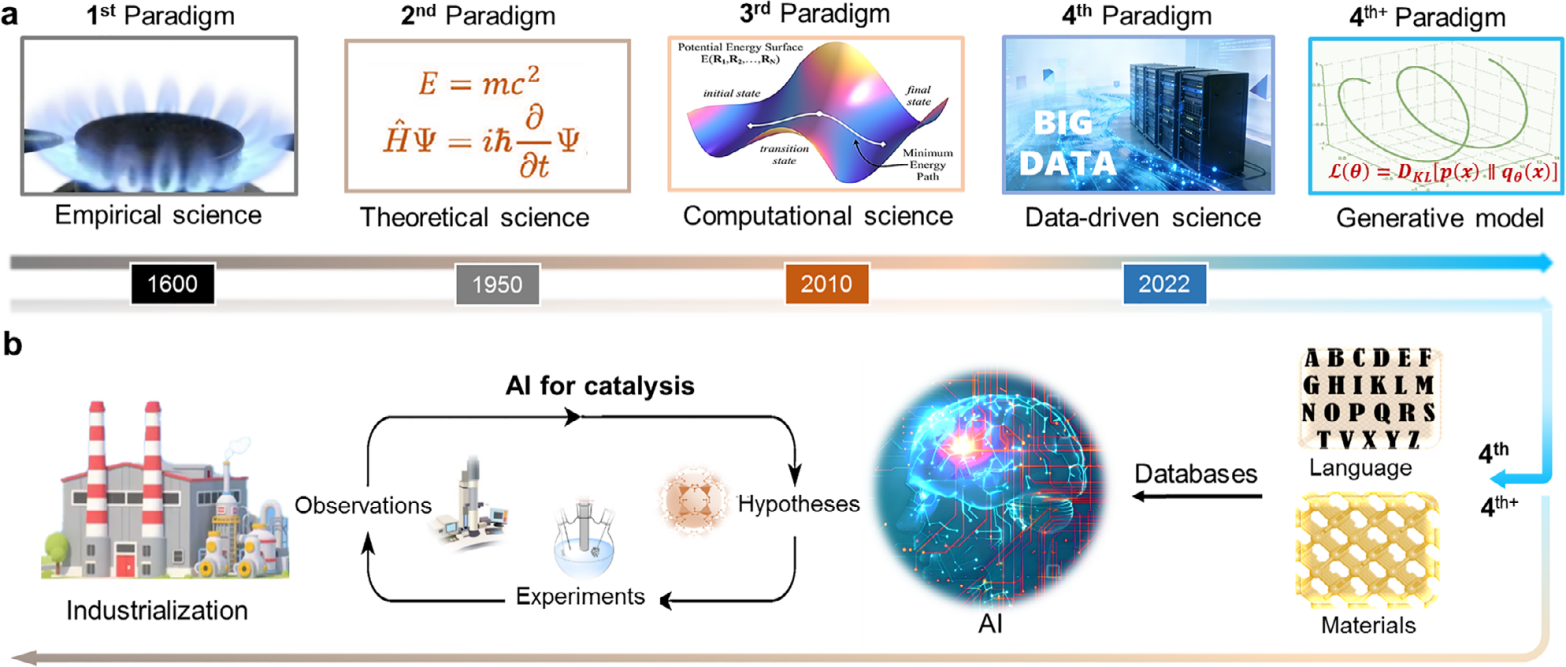
Catalysis research paradigms. (a) Four research paradigms of catalysis: empirical science (first paradigm), theoretical science (second paradigm), computational science (third paradigm), and data‐driven science (fourth paradigm), in which the generative model is considered as the fourth^+^ paradigm. The identifier of potential energy surfaces was reproduced with permission from Ref. [4]. Copyright 2015, IOP Publishing Ltd. (b) The fourth/fourth^+^ paradigm integrates databases, physics‐based models, and agentic labs for autonomous catalyst discovery and accelerating the catalytic process of industrialization. The identifier of industrialization was reproduced with permission from Ref. [9]. Copyright 2025, American Chemical Society.

Catalysis underpins modern society, enabling key processes in agriculture, energy, and environmental protection [1, 2, 3, 4]. The design and discovery of catalyst materials are driving forces for accelerating scientific and technological innovations in the fields of energy conversion, environmental remediation, and chemical industry. The progression of catalyst development strategies embodies a fundamental evolution across four distinct research paradigms (Figure 1a): empirical science, theoretical science, computational science, and data‐driven science. This evolution began with trial‐and‐error experimentation, which yielded fragmented datasets but established foundational concepts such as the seminal *Sabatier* principle [5]. The advent of quantum chemistry and density functional theory (DFT) in the 20^th^ century marked a paradigm shift toward theoretical and computational sciences, enabling atomic‐scale insights through theoretical descriptors such as the *d*‐band center and adsorption energies [6, 7]. While DFT bridged electronic structure with macroscopic performance and generated essential catalyst data, its limited length and time scales, combined with difficulties in handling complex interfacial and disordered structures, have led to a persistent “complexity gap” between theory and experiment. Entering the 21^st^ century, the exponential growth of experimental data and computational power has positioned data‐driven science at the forefront of catalysis research, driving the emergence of big data analytics and data‐driven methodologies [8, 9]. Generative models can be viewed as an extension of the data‐driven paradigm (here, termed the fourth^+^ paradigm) [10]. What fundamentally distinguishes them is not their inherent nature as data‐driven processes, but rather the scale and methodological sophistication through which they generate, process, and extract knowledge from data.

Ushering in the driven science paradigm, artificial intelligence (AI) is emerging as a powerful enabler, offering transformative forces to drive the advancements in the field of catalysis science (Figure 1b). With the rise of universal machine learning interatomic potentials (MLIPs) and large language models (LLMs) [8, 11, 12] in recent years, these cutting‐edge AI technologies create strong demand for large, high‐quality datasets, offering unprecedented opportunities to accelerate catalyst discovery and development. By achieving near‐DFT‐level accuracy with orders‐of‐magnitude speedups over large‐scale structure models, universal MLIPs bridge the gap between quantum‐mechanical precision and large‐scale exploration of catalyst materials, thereby overcoming the computational limitations of conventional DFT‐based methods [13, 14]. The methodology of LLMs involves training neural networks on vast amounts of text data, enabling them to understand, generate, and reason about human language [15], demonstrating immense potential for catalytic text‐based knowledge extraction from scientific publications. The integration of AI‐driven methodologies (universal MLIPs + LLMs) would enable a series of data‐centric catalysis platforms and databases [16, 17], a key ingredient for further training reliable data‐driven predictive models. Their applicability allows researchers to construct catalysis platforms, analyze complex datasets, identify hidden patterns, and predict catalytic properties with remarkable precision. Additionally, these AI technologies, combined with agentic labs (i.e., autonomous by robotic agent), will improve the efficiency of data preparation and model development, enabling a seamless integration of computation, data analytics, and catalyst design [18].

As a forward‐looking Mini‐Review, we start the journey by exploring the transformative role of databases and AI × databases in catalysis, emphasizing the potential of universal MLIPs and LLMs to revolutionize catalyst design, and then review their applications and capability to deliver highly accurate predictions in the catalysis field, as well as discuss the existing challenges in catalysis research. To efficiently leverage these cutting‐edge AI technologies, several outlook approaches are proposed, such as AI Closed‐Loop and Digital Materials Ecosystem, which are discussed to pave the way toward more intelligent, data‐driven catalyst design and discovery.

## 2 Database for Catalysis

### 2.1 Database for Catalysis Research

**FIGURE 2:**
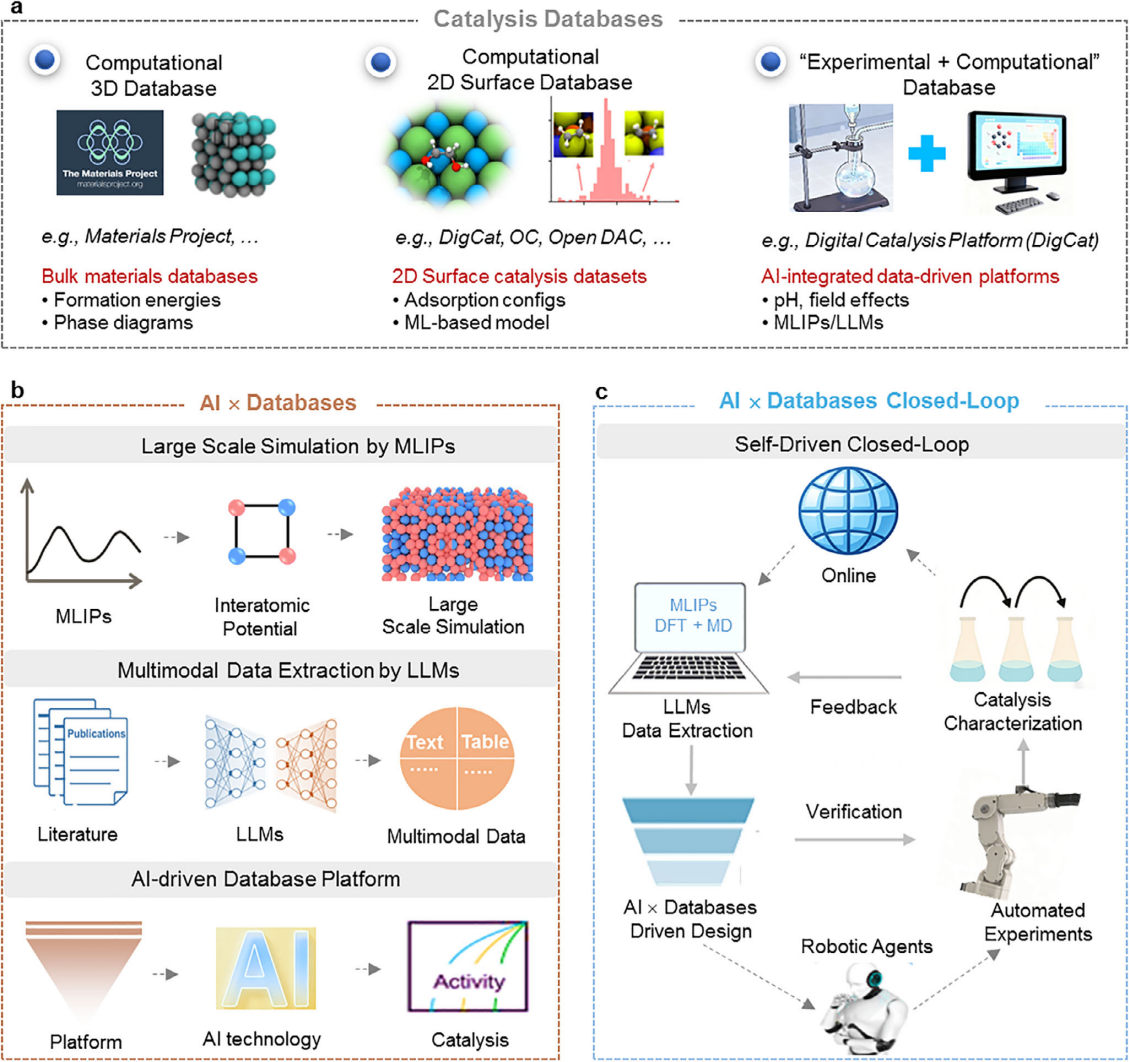
Database for catalysis science. (a) The catalysis databases evolve from bulk materials databases to AI‐integrated platforms. Some identifiers were reproduced from the Materials Project database, licensed under CC BY 4.0, and Ref. [22]. Copyright 2021, American Chemical Society, respectively. (b) The AI × database, driven by universal MLIPs and LLMs, toward intelligent catalysis database platforms. (c) The AI × database closed‐loop for catalysis.

The dramatic growth of computational resources has catalyzed the emergence of large‐scale materials databases that are fundamentally reshaping catalyst discovery. In recent years, the landscape of computational 3D structure databases, computational 2D surface databases, and “experimental + computational” databases has expanded substantially (Figure 2a), collectively providing up to tens to hundreds/millions of catalyst‐associated data points across diverse material classes. Foundational repositories like the Materials Project [19], housing over 154,000 materials with systematically computed formation energetics and corresponding phase and Pourbaix diagrams, enable rapid screening of thermodynamic stability under operating conditions. Based on developing volcano models by DFT‐based calculations and capturing the role of solvent effects by molecular dynamics (MD) simulations, the Materials Project can be used to understand and accelerate catalytic materials discovery through high‐throughput computation. Several 2D surface catalysis databases have been built such as Digital Catalysis Platform (*DigCat*, www.DigCat.org) [20], Catalysis‐Hub (www.catalysis‐hub.org) [21], the Open Catalyst Project (OC20 [22]/OC22 [23]/OC25 [24], opencatalystproject.org), and Open DAC 2023 (open‐dac.github.io) [25]. In comparison, databases such as Materials Project, OC20/OC22/OC25, and Catalysis‐Hub are predominantly theory‐driven and mainly consist of idealized structures and their corresponding computational properties. Materials Project primarily focuses on bulk material calculations, whereas catalysis research critically depends on surface structures and surface‐related properties, such as adsorption energies and reaction intermediates. Databases such as Catalysis‐Hub, OC20/OC22/OC25, and *DigCat* therefore place greater emphasis on surface models and adsorbate–surface interactions. ODAC23 is mainly designed for direct air capture applications, involving metal–organic frameworks (MOFs) with CO_2_ and H_2_O adsorbates. Through these initiatives, the landscape of surface property databases has expanded remarkably, collectively providing millions of adsorption energies across diverse material classes from metallic surfaces to MOFs. In particular, the *DigCat* integrates both computational and experimental data, offering over 900,000 data entries that reveal previously inaccessible trends, such as pH‐dependent activity in M–N–C electrocatalysts that have challenged existing theoretical frameworks [20], and decodes pH‐dependent electrocatalysis through electric‐field models and microkinetic volcanoes. These databases serve as foundational pillars for AI‐driven exploration of catalysis and foster collaboration and knowledge sharing within the global catalysis community. Seamless integration between databases from different disciplines will enable cross‐domain research, supporting the broader application of AI chemists in catalyst design and synthesis.

Data collection and analysis are fundamental to understanding catalytic correlation effects and ultimately to discovery. The quantity, quality, and precision of data play a crucial role in determining the types of questions that can be addressed through data science. High‐quality, comprehensive databases are widely recognized as a foundation for data‐driven catalyst design. A high‐quality training dataset for AI models should be homogeneous, sufficiently large, and representative, and a sufficiently comprehensive dataset is necessary for the model to capture the underlying catalytic phenomena. These requirements require our efforts to improve the libraries of catalyst materials, reactions, parameters, force fields, *etc*., ensuring data accuracy, consistency, and standardization, and to collect data from *operando* experimental conditions, high‐accuracy DFT computational tools, and literature data mining. A key bottleneck is standardization of critical details on material identity, synthesis, characterization, reaction conditions, and measurement protocols are often missing or lost during extraction, which limits cross‐dataset comparability, model generalization, and the reliability of AI‐driven predictions.

### 2.2 AI × Database Platform for Catalysis

Accompanying the rapid pace of catalysis development is a pressing need from the wider research community to establish a high‐quality, scalable, and intelligently recommended database platform. For instance, the established *DigCat* platform is a gateway to big data and AI‐powered innovations in catalysis, which can serve as a pivotal platform for the experimental and computational catalysis community [20]. It supports data ingestion, analytics, benchmarking, and LLM‐assisted query and modeling.

With ongoing advances in universal MLIPs and LLMs, there is a promising trajectory for integrating these AI approaches into catalysis data platforms (Figure 2b). The MLIPs are constructed from data generated by DFT calculations, with total energies and atomic forces as training targets [26]. By learning the relationship between local atomic environments and these properties, MLIPs can accurately reproduce potential energy surfaces (PES). It has emerged as a transformative approach, bridging the gap between quantum mechanical precision and large‐scale exploration of catalyst materials, enabling efficient large‐scale simulations [27]. LLMs have demonstrated immense potential for catalytic text‐ and table‐based knowledge extraction from scientific publications. LLM‐powered literature mining exhibits greater adaptability and generalizability due to the natural language processing, conversational capabilities, and general‐purpose versatility of LLMs. In particular, the emergence of multimodal LLMs [28, 29, 30], which can receive and process multiple data modalities, including images, text, audio, and others, offers a more comprehensive approach to data mining. Some of these multimodal LLMs have reported promising accuracies on various graph/text‐language multimodal tasks, such as image captioning and text understanding in generic everyday publications, across different evaluation benchmarks [30]. By synergizing MLIP data with LLM text mining, it is expected to enhance database quality and significantly accelerate catalyst discovery and application, ultimately building a smarter, open‐access, and dynamically updated “AI × database” platform.

We suggest integrating catalysis databases into an overarching, curated platform and diversifying its coverage through three distinct steps. In the first step, data collection and integration are required to consolidate diverse experimental and computational catalysis databases, standardize representation formats, and augment key properties through computational supplementation. Meanwhile, we need to continuously expand the dataset through MLIP calculations and LLM‐based text mining of literature. The second step of data correction and management is crucial, comprising deduplication and error correction to eliminate redundancies/biases that may interfere with model training. At this point, a possible path forward is to leverage LLMs, whose collective knowledge of large databases can help correct errors in individual entries. In data management, we can employ ensemble machine learning models to perform cross‐validation‐based property predictions and establish expert‐review protocols for entries with prediction discrepancies. The third step is to push data diversification and expansion. Increasing the volume of DFT data does not necessarily improve model performance. Instead, computational datasets should be constructed to systematically and efficiently cover the relevant configurational and chemical phase space. In addition, experimental performance is strongly influenced by real material composition, structural heterogeneity, morphology, and testing conditions, which are often simplified or absent in computational models. Therefore, we also emphasize the need for robust experimental data platforms, which can serve as a reliable benchmark to validate physics‐based catalytic models and provide the foundation for training deep learning models that incorporate these experimentally relevant factors.

### 2.3 AI × Database Closed‐Loop for Catalysis

Recent advances in LLMs for catalytic data mining have significantly impacted catalysis science, particularly in autoregressive generation, data retrieval, and autonomous catalyst discovery. Farimani et al. [31]. extend this success to the catalysis domain, particularly by enhancing the predictive capability of transformer‐based language models for adsorption energy, an essential quantity for screening catalysts. They developed a graph‐assisted pretraining approach, an innovative multimodal learning framework that utilizes graph representations to enhance the language model's predictive capability for adsorption configurations. Through self‐supervised learning, this method effectively transfers structural knowledge encoded in graph embeddings to corresponding text embeddings. Such cross‐modal knowledge transfer significantly improves the model's performance in predicting adsorption configuration energies, which is particularly valuable for multimodal LLMs’ specific applications.

Moving forward, with the advancements in LLMs, the expansion of databases, and the development of automated synthesis technologies, we can develop a more comprehensive, language‐based platform for catalyst design and/or a closed‐loop AI‐driven catalyst synthesis system, and equip the platform/system with reasoning and planning capabilities in an Agent‐like framework (Figure 2c). By integrating with the LLMs technologies, the closed‐loop AI‐driven framework can contain the following route: based on actual demand, the LLMs can access sub‐libraries and literature database containing information about reactions, materials, and synthesis methods to design the potential catalysts and generate the initial plan for synthesis. Subsequently, high‐throughput robotic systems execute the plan to produce candidate catalysts, which are then characterized in a high‐throughput manner to evaluate their catalytic performance and structural properties. The characterization results are fed back into the ML algorithms to optimize the synthesis plan further. After several iterative processes, the desired catalyst is finally obtained.

In machine‐readable markup languages, data modalities often pose unique challenges. Analyzing and converting complex structures, such as large tables or plots, is usually tricky. This problem is becoming increasingly prominent in the extraction of catalytic data, where, in addition to plots and tables, crystal structures, reaction schemes, complex spectra, and intricate graphical representations contain vast amounts of critical information. To address these issues, multimodal LLMs are expected to understand and analyze images alongside text. For instance, the recently developed vision language models (VLMs) [29, 30, 32] can be helpful in extracting useful information from diverse types of data one might encounter in scientific literature, such as tables, formulas, structure files (*e.g*., CIF files for crystals), and sub‐figures containing images with intricate relationships. These advancements create further opportunities to make data extraction more robust and accessible, which we describe across several research frontiers.

A fundamental challenge may stem from the multimodal nature of catalytic information, in which key relationships may be distributed across different representation formats; a typical example is tabular property data linked to graphical elements *via* document cross‐references. While current parsing approaches produce structured outputs (*e.g*., LaTeX and XML), these outputs may lack the optimal organization required for practical training of machine learning models. This highlights the necessity of next‐generation LLM tools that can produce structurally diverse, machine learning‐friendly representations of extracted scientific knowledge. Future work remains to make them more robust and adaptable to the diversity of data found in catalysis science. With the development of large models that will further enhance the completeness of catalytic data, there are vast opportunities to refine and expand data acquisition technologies, thereby advancing the field of data‐driven catalysis.

Building on this integrated vision, the following discussion examines the respective roles and recent advances of two core technological enablers: MLIPs and LLMs. We will first discuss how MLIPs (particularly universal MLIPs) are transcending the accuracy‐efficiency trade‐off to enable high‐fidelity, large‐scale catalytic simulations. Subsequently, we will explore the emerging capacity of LLMs to navigate the knowledge‐experiment continuum, from semantic understanding of literature to the orchestration of self‐driving laboratories. This bifurcated analysis aims to provide a granular understanding of how each component uniquely contributes to reshaping the catalysis research paradigm.

## 3 Universal MLIPs for Catalysis

### 3.1 Definitions and Conceptual Frameworks

**FIGURE 3:**
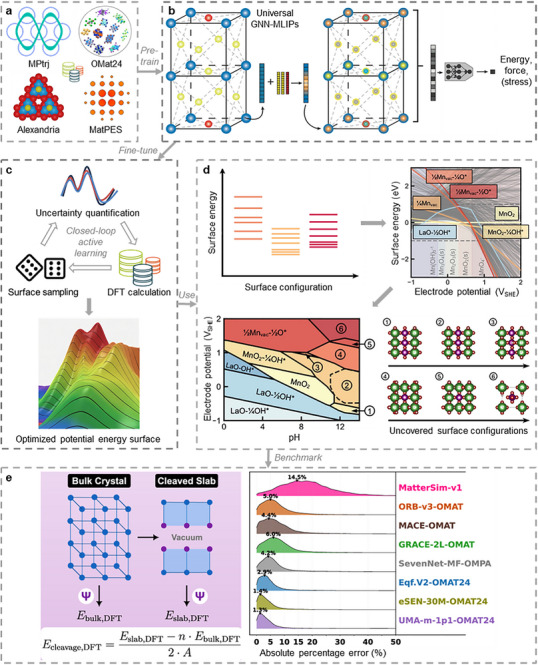
Catalyst materials design by universal MLIPs. (a) Universal MLIPs are generally trained on large‐scale datasets of DFT trajectories (*e.g*., energy and forces during structural relaxation, *ab initio* MD, and structural sampling), with representative datasets including MPtrj [14], OMat24 [44], Alexandria [45], and MatPES [46]. (b) GNN architectures are commonly used to build MLIPs because they can naturally interpolate the PES to infer energy, forces, and stress by capturing the coordination environment of each atom in a crystal structure *via* message passing and graph convolutions between nodes (atoms) and edges (bonds) in the graph representation. Adapted from Ref. [47]. Copyright 2023, Annual Reviews. (c) Active‐learning loops fine‐tune universal MLIPs efficiently using uncertainty‐driven data acquisition. (d) By leveraging prior knowledge in pre‐trained universal MLIPs and fine‐tuning these models with only 136 additional DFT calculations, a thoroughly sampled Pourbaix diagram of the LaMnO_3_(001) surface can be generated for dissolution and reconstruction, uncovering two new surface states that have not been reported. La, Mn, O, and H atoms are shown as green, purple, red, and white spheres, respectively. Reproduced from Ref. [48]. Copyright 2025, American Chemical Society. (e) Comparative performance analysis of eight state‐of‐the‐art universal MLIPs for cleavage energy prediction on a high‐throughput DFT database of 36,718 surface structures spanning elemental, binary, and ternary metallic compounds. Reproduced from Ref. [49]. Copyright 2025, IOP Publishing Ltd.

MLIPs are shifting atomistic modeling from problem‐specific force fields to general‐purpose interatomic models applicable to catalysis (Figure 3a) [33, 34, 35]. MLIPs serve as efficient surrogate models for the PES with near‐DFT accuracy. MLIPs predict energies and forces from atomic positions, enabling much faster simulations of atoms, solids, and interfaces. Traditional MLIPs include the Behler‐Parrinello neural networks [36], which utilize handcrafted symmetry functions, and Gaussian approximation potentials [37], which employ descriptors such as atomic cluster expansion. These conventional methods capture local environments using fixed functional forms with human‐designed descriptors. In contrast, modern MLIPs leverage graph neural networks (GNNs), which represent atoms as nodes and bonds as edges [38] (Figure 3b). Through iterative message passing among neighboring atoms, GNN‐based MLIPs learn embedded representations and many‐body atomic environments without manual feature engineering [39], enabling expressive modeling of materials [40]. Graph‐based learning yields higher accuracy and transferability across chemical space than descriptors, as demonstrated by GNN models such as SchNet [41] and DimeNet [42]. Recent advances that incorporate attention mechanisms and equivariant layers [43] for rotational symmetries further improve performance.

Universal MLIPs are trained on large, diverse datasets spanning many configurations [50], rather than being specialized to a narrow chemical domain. They aim for broad coverage with various structures, making them attractive starting points for exploring unknown chemistries. Notable examples include M3GNet [51] and CHGNet [52], which were trained on a decade of DFT relaxation trajectories generated by the Materials Project [19]. These models encompass dozens of elements and have demonstrated predictive capabilities across diverse crystals [53] and surfaces [49, 54] without extensive fine‐tuning. Trained on large, relaxed‐structure datasets, they transfer rather well to related systems and remain relatively stable in MD simulations [55] across various materials. Universal MLIPs can serve as general‐purpose DFT surrogates for materials exploration with near‐DFT accuracy for a given functional (*e.g*., the Perdew–Burke–Ernzerhof functional, PBE).

Here, we distinguish “universal MLIPs” from “foundation models” by the scope of their downstream capabilities rather than by dataset size or chemical space coverage alone. A foundation model is a large‐scale model pre‐trained on diverse data (Figure 3a) that can be fine‐tuned for many downstream tasks [56]. By contrast, a universal potential may cover broad chemistry but is typically trained for a single purpose (*e.g*., predicting PBE energies) and has not yet demonstrated broad task adaptability. For example, MACE‐MP‐0 [57]—trained on the Materials Project Trajectory (MPtrj) Dataset [14]—achieves excellent accuracy and stability across many crystals and functions as a universal PBE potential. Still, its ability to fine‐tune for unrelated tasks remains unproven. In contrast, it has been recently proposed [58] that true foundation models are expected to adapt across different levels of quantum theory, predict properties beyond energies (*e.g*., charges, barriers, and field responses), generalize to out‐of‐distribution scenarios after fine‐tuning, and outperform task‐specific models across diverse downstream simulation tasks. Thus, we use universal MLIPs to denote broadly pretrained PES surrogates (typically trained to reproduce a single level of theory, such as PBE energies/forces) that provide strong out‐of‐the‐box coverage across many elements and structures, but whose transfer to new tasks often requires careful domain‐specific adaptation. By contrast, foundation models denote pretrained models explicitly designed for systematic fine‐tuning across diverse downstream objectives, such as adapting across levels of theory and predicting richer observables beyond energies/forces (such as charges, response properties, and/or reaction barriers), with the expectation of improved data‐efficiency and cross‐task transfer after fine‐tuning. To clarify, instead of “foundation MLIPs,” we use “universal MLIPs” to refer to state‐of‐the‐art GNN‐based MLIP models with comprehensive element coverage, diverse training data, and a general PES that can be refined for specific tasks. The term “universal” highlights training on massive, multi‐element datasets and applicability across various materials. Developing such models requires universality in the input domain and support for fine‐tuning, transfer learning, and knowledge distillation. In catalysis, this means handling metals, semiconductors, alloys, and adsorbates, ideally incorporating all catalytic elements. The following sections discuss how such models are trained, their emerging applications in heterogeneous catalysis, benchmarking practices, and remaining challenges.

### 3.2 Training Paradigms and Data Infrastructure

A strategy toward universal MLIPs involves pre‐training GNNs on broad datasets, refining them on narrower tasks *via* fine‐tuning (Figure 3b). For example, a model can be first trained on a wide, lower‐level DFT dataset to learn general chemical trends, and then further trained on a smaller, task‐specific or high‐accuracy dataset to yield refined PESs [59]. This multi‐step training reduces the required data volume because the model already encodes fundamental chemical properties, such as bond fingerprints [60]. Recent studies show that fine‐tuning universal MLIPs improves accuracy for specific targets compared to training models from scratch [61]. Similarly, multi‐fidelity training [62] and transfer learning [63] accelerate convergence and enable knowledge transfer across different environments. Moreover, delta learning is another technique in which MLIPs learn the difference between two levels of theory or between a cheap baseline and a target outcome, thereby generating a machine‐learning‐predicted correction term for a fast, accurate interatomic potential. In catalysis, delta‐learning could involve solvation effects by learning corrections on top of simpler models [64]. Furthermore, modern MLIPs could have hundreds to millions of parameters, prompting interest in model compression *via* knowledge distillation. Here, an ensemble of huge teacher models generates additional training data or gradients for a smaller student model, which learns to mimic the teacher's predictions. This yields a more compact model with near‐original accuracy, faster inference, and lower memory usage, making it useful for long MD simulations [65] or lightweight deployment on hardware [66].

Pre‐training and fine‐tuning universal MLIPs are challenging due to the vast chemical space; therefore, active learning prioritizes which configurations to compute with DFT and add to the training set. For example, GNoME iteratively refines its model by predicting new stable structures, validating them with DFT, adding them to the dataset, and retraining in a closed cycle (Figure 3c). Uncertainty quantification (UQ) is crucial in this cycle: helping the model recognize when it is extrapolating beyond its domain. Techniques such as ensemble predictions [67], adversarial attacks [68], and test‐time refinement [69] identify high‐uncertainty, out‐of‐distribution predictions for DFT evaluation, preventing the model from overlooking poor‐performance regions. UQ also supports safe deployment by indicating uncertainty during long MD simulations when trajectories drift far outside the training domain, prompting corrective DFT calls or avoiding unreliable regimes [70, 71]. MD simulations at high temperature or involving catalytic reactions can generate configurations absent from the training dataset, potentially causing unphysical force spikes that “blow up” the simulation [72]. To mitigate this, some training regimens include random perturbations and far‐from‐equilibrium structures, teaching the model to avoid failures [73]. Additionally, enforcing physical constraints [74] further improves robustness outside the domain. Ultimately, an ideal universal potential should handle extreme atomic arrangements gracefully rather than produce erratic forces [75].

Most current MLIPs are trained solely on DFT energies and forces from static calculations or short trajectories, thereby omitting higher‐order information, such as Hessians, that describe the local PES shape. As second derivatives of energy, Hessians are related to vibrational frequencies and the curvatures of PESs; incorporating Hessians or vibrational modes can yield smoother, more accurate landscapes near equilibrium [76, 77]. Prior work shows that including force constants improves phonon properties and entropic predictions in catalysis [78]. Another consideration is long‐range interactions, as short‐range cutoffs (5‐8 Å) miss essential physics like electrostatics or dispersion. Some models address this by incorporating Coulombic interactions (*e.g*., predicting partial charges [79] or multipole moments [80] that are connected to Ewald summation) [81]. Furthermore, incorporating charge equilibration, polarizable terms, or many‐body dispersion corrections extends the applicability of MLIPs to surfaces and interfaces where long‐range fields are essential, such as charged electrocatalytic interfaces or physiosorbed reaction intermediates [82].

A significant advance in data‐efficient MLIPs is the development of rotation‐equivariant MLIPs (*e.g*., NequIP [83] and MACE [84]). Equivariance ensures that model features transform predictably under rotations or reflections, preserving tensor properties rather than forcing the model to learn symmetry by brute force [85]. Embedding such symmetries reduces the training data [86], as the network does not have to explicitly “learn” basic invariances [87], essential for complex atomistic systems like reactive surfaces [88]. Therefore, the equivariant models perform well in data‐sparse regimes that incorporate expensive DFT data. Moreover, symmetry‐aware MLIPs better capture chemical disorder, improving predictions for complex systems such as multicomponent metal oxides [38, 89, 90] and high‐entropy alloys [91]. However, recent studies show that optimized non‐equivariant [92] and even non‐energy‐conserving [93] architectures can achieve accuracy comparable to their equivariant, energy‐conserving counterparts. This challenges the assumption that strict equivariance or energy conservation is always necessary for accuracy, suggesting that sufficiently large datasets can allow simpler models to learn complex physics implicitly. Nonetheless, most materials lack massive datasets; leveraging symmetry equivariance remains the most reliable strategy for improving data efficiency and reliability of MLIPs.

In summary, training universal MLIPs is shifting from supervised learning on fixed datasets to a multi‐stage workflow: large‐scale pre‐training to learn universal patterns; fine‐tuning to reach target accuracy on specific tasks; and continuous active learning to expand coverage. Incorporating UQ, adversarial cases, and richer training labels (*e.g*., stresses, Hessians, and charges) further strengthens model robustness. Such rigorously trained MLIPs are better equipped to handle the complexities of catalysis simulations, as discussed next.

### 3.3 Application to Heterogeneous Catalysis

Heterogeneous catalysis is challenging due to dynamic surface reconstruction, active‐site formation, and the effects of solvents or electrolytes. Universal MLIPs are increasingly enabling large‐scale, realistic simulations previously infeasible with DFT.

Many catalysts (*e.g*., late transition metals) exhibit dynamic surface structures that respond to adsorbates, temperature, and electrochemical potential. MLIPs can capture segregation, facet reconstruction, and roughening; for example, MLIP‐powered MD has achieved microscale surface simulations involving nearly trillions of atoms [94] and has been used to model the evolution of Cu oxides to oxide‐derived nanocrystalline Cu, clarifying the active sites for CO_2_ reduction [95]. Moreover, MLIPs combined with grand canonical Monte Carlo can unbiasedly explore the reconstructed surface of SrTiO_3_(001) using ∼5,000 single‐point DFT calculations [96]. Likewise, fine‐tuning pre‐trained universal MLIPs with only 136 additional structures reproduced the surface Pourbaix diagrams of LaMnO_3_(001) dissolution and reconstruction, and revealed two previously unknown terminations (Figure 3d) [48]. MLIPs can further capture dynamic active site formation, such as the mesoscopic surface reconstruction on Au [97] and kinetic timescales of low‐barrier surface reconstruction on O‐covered Pd surface [98]. These capabilities matter as the active state of a catalyst may be a transient structure and inaccessible to static DFT or *ab initio* MD (AIMD) simulations. Universal MLIPs (capable of sampling nanosecond‐scale dynamics across large supercells) reveal which surface configurations are populated and active in *operando* conditions.

MLIPs are also used to calculate accurate reaction free energy landscapes on catalyst surfaces. Coupling MLIPs with sampling techniques such as umbrella sampling and thermodynamic integration enables the precise computation of free energy barriers and rate constants. MLIP‐driven MD also captures anharmonic effects in reaction kinetics. For instance, an MLIP study of NO desorption from Pd(111) demonstrated that including lattice vibrations and anharmonicity accurately predicts desorption rates and prefactors beyond harmonic transition‐state theory [99]. By employing extensive sampling, MLIPs yield entropy and enthalpy contributions along reaction coordinates, advancing catalysis modeling from static PESs to full finite‐temperature free energy surfaces [100]. Their fast force evaluations further accelerate transition state search [101], reaction dynamics exploration [102], and mechanistic modeling [103] across complex reaction networks on catalysts (*e.g*., parallel reaction pathways and intermediate pools).

MLIPs are advancing in electrocatalytic systems that introduce electrolytes and applied electrode potentials. While incorporating an explicit electric field remains challenging, since most models are trained on neutral systems, solvent‐inclusive MLIPs have enabled realistic simulations of solid–liquid interfaces. For example, MLIPs trained to describe water on Ru(0001) enable MD simulations of an aqueous interface and solvation structures [104]. Similar simulations of water on Cu(111) investigated how water layers order and affect adsorption sites, revealing hydrogen‐bonding networks and dynamics that are inaccessible to traditional AIMD. Moreover, MLIPs that capture long‐range electrostatics are essential for predicting dielectric responses and electronic polarization at electrocatalytic interfaces [105]. Achieving such transferability requires training datasets that span ionic compositions, proton activities, and interfacial electric fields (*e.g*., the OC25 dataset [24] as a recent example). MLIPs can also support quasi‐static modeling of proton‐coupled electron transfer by sampling solvated proton‐transfer configurations and evaluating their energetics with a pre‐trained or refined potential. Ideally, charge‐aware MLIPs can accelerate the modeling of electrocatalyst interfaces by predicting electric‐field‐dependent surface energetics or by coupling with constant‐potential simulations. Despite this progress, the MLIP‐powered modeling of electrocatalytic systems remains far from a solved problem because the electric double layer and solvent structure are strongly coupled and evolve dynamically with potential, ionic strength, and surface charge [106]. In practice, most current interfacial MLIP studies still rely on approximations such as fixed‐charge slabs, limited ion speciation, short time windows, or sampling that does not fully capture rare but catalytically relevant restructuring events. The key observables of the reaction interfaces (*e.g*., potential‐dependent surface charge, local electric field, and ion adsorption) are likely not fully treated by existing MLIPs unless charge transfer and long‐range electrostatics are modeled consistently, which challenges traditional short‐range GNNs. These limitations imply that even the best “explicit solvent electrochemical modeling” in today's MLIP‐driven simulations often provides a structurally resembling hydration environment but not yet a fully predictive description of potential‐controlled thermodynamics and kinetics for modeling electrochemical interfaces.

In conclusion, universal MLIPs can help enable realistic catalysis simulation—high coverage, finite temperature, multi‐component systems, and explicit solvent—with near‐DFT accuracy. They bridge the gap between static, idealized DFT calculations and the dynamic, disordered environments of catalysts. By capturing surface and reaction dynamics, MLIPs reveal mechanistic factors that govern catalytic performance. The following section discusses how such models are benchmarked, validated, and assessed for generality.

### 3.4 Leaderboards and Benchmarking Efforts

Benchmarking has become essential for fair comparison and driving the progress of MLIPs. Several large‐scale benchmarks with distinct focuses now guide the materials communities. Matbench Discovery [107] is a recent framework designed to evaluate MLIPs for discovering new stable materials. Instead of simple regression error, it measures how effectively a model can pre‐filter vast candidate pools to identify stable crystals without DFT. Universal MLIPs, such as eSEN [26] and Nequip‐OAM‐L [108], currently lead this benchmark. Many material properties (*e.g*., lattice vibration, atomic diffusion, and chemical reactions) depend on finer aspects of the PES, which are not captured by the metrics in the Matbench Discovery. To partially address this issue, lattice thermal conductivity [109] was recently incorporated to provide a stricter test of PES smoothness. To further overcome issues with existing benchmarks, including data leakage, limited transferability, and DFT‐centric errors, the newly introduced MLIP Arena [110] evaluates physics‐awareness, reactivity, and extreme‐condition stability. By exposing real‐world failure modes that static tests miss, MLIP Arena enables more transparent benchmarking and guides the development of physically consistent MLIPs.

While leaderboards are helpful, they also have limitations. Many benchmarks rely on static error metrics (*e.g*., MAE and RMSE), but low test error doesn't always guarantee better performance in practical simulations. One model with slightly higher MAE may still be reliable in MD if its errors do not contain catastrophic outliers. Another limitation is that many benchmarks test interpolation, not real extrapolation: the test data often come from the training distribution. In catalysis, however, MLIPs frequently encounter entirely new materials, temperatures, or surface coverages [111]. Recent evaluations of MACE, CHGNet, and M3GNet on out‐of‐the‐box surface energy showed significant errors correlated with how dissimilar the surfaces were from bulk configurations in the training set, exposing an extrapolation gap and highlighting that leaderboards could overestimate real‐world robustness [49].

Additionally, benchmarks often emphasize single‐number metrics over physical criteria. A model might win on RMSE but violate conservation laws. More physics‐based tests are emerging: for example, eSEN [26] introduced an NVE MD test on an out‐of‐distribution system to assess energy conservation. Models that passed this test also showed improved property prediction. Lastly, current leaderboards rarely account for long‐term or multi‐step predictions. Catalysis is inherently a time‐integrated process: small force biases can accumulate over nanoseconds, leading to qualitatively incorrect behavior (*e.g*., a molecule diffuses away instead of remaining adsorbed). Developing metrics that measure trajectory drift relative to AIMD, or the ability to reproduce kinetic rates and rare‐event dynamics, remains an essential but underexplored frontier.

Surface‐ and interface‐focused benchmarks are also emerging because they emphasize precisely the out‐of‐distribution regimes in which universal MLIPs most often fail in practice. However, compared with bulk‐property leaderboards, large‐scale surface‐ and interface‐focused benchmarks of universal MLIPs remain relatively rare at present. A recent cleavage‐energy benchmark [49] illustrates this point by evaluating multiple state‐of‐the‐art universal MLIPs against a high‐throughput database of 36,718 slab structures spanning elemental, binary, and ternary metallic compounds, where the target property is explicitly surface‐dominated and therefore less aligned with bulk‐heavy training distributions. Notably, the error distributions across representative models show substantial spread and pronounced tails rather than small, uniformly distributed deviations, indicating that leaderboard‐style average metrics can mask rare but consequential failure modes when models are deployed for surface stability screening or catalytic interface modeling (Figure 3e). By complementing bulk‐centric leaderboards with such surface‐specific tests, benchmarking can better diagnose whether a seemingly well‐performing universal MLIP is merely interpolating within familiar coordination environments or is robust enough for the low‐coordination, reconstruction‐prone conditions that dominate catalytic operation.

More broadly, despite impressive progress in accelerating simulations and reproducing known structure–property trends, prospective end‐to‐end demonstrations in which a universal MLIP directly enables the experimental synthesis of a genuinely new, performance‐leading catalyst remain rare, highlighting a persistent ML–theory–experiment gap. This gap largely reflects the difficulty of reliable extrapolation to new chemistries and *operando* states, underscoring the need for uncertainty‐aware workflows and tighter closed‐loop integration with experiments [112].

Moving forward, more comprehensive benchmarks, including dynamic simulations, stress tests, and application‐oriented tasks (*e.g*., complete catalytic cycles or deactivation pathways), will be essential for guiding the next generation of universal MLIPs.

### 3.5 Existing Challenges and Future Direction

Despite significant progress, several challenges remain before universal MLIPs become routine tools for catalyst discovery and mechanistic studies. First, universal MLIPs need broader, diverse, and higher‐fidelity training data. This includes surface and interfacial configurations, nanoparticle structures, materials, electrolyte environments, and related parameters. Current models favor bulk‐like structures, leading to transferable gaps at surfaces and in low‐coordination environments. Moreover, higher‐level data and cross‐domain transfer training techniques (*e.g*., hybrid functionals and experimental data integration) still require further optimization and implementation to correct the systematic errors in baseline DFT calculations. Approaches such as fine‐tuning with small, high‐quality datasets or multi‐fidelity training (using inexpensive data for trends and costly data for details) could achieve chemical accuracy at scale. For example, the MatPES project's release of ∼400k static DFT calculations, including consistent PBE and r^2^SCAN data for training universal potentials [45]. Additionally, MP‐ALOE [113], a nearly 1 million–structure dataset computed with r^2^SCAN and active learning with mainly off‐equilibrium structures across 89 elements, enables MLIPs to accurately predict properties, far‐from‐equilibrium structures, and MD behavior under extreme conditions, providing a resource for multi‐fidelity training. In the future, we need to build universal models trained on a standardized, high‐quality, cross‐domain dataset, with varying levels of accuracy.

In addition, despite being data‐driven, top MLIPs should embed more physics to ensure accuracy. One approach is to enforce energy conservation, with many models deriving forces from the gradients of a single energy function; however, newer models have relaxed this constraint. Ultimately, for reliable MD over long times, the potential should be conservative. Future architectures need to balance strict conservativeness with expressivity. Equivariance is another physics prior, offering efficient capture of angular forces at modest cost. Second, long‐range electrostatics and charge response are a particularly severe bottleneck for MLIP‐based modeling of electrocatalytic systems because most GNN‐based MLIPs use finite‐cutoff message passing and therefore remain effectively local. Consequently, many “universal” MLIPs struggle to account for nonlocal phenomena such as charge redistribution, long‐range polarization, and electrostatic screening, even though these effects often govern adsorption energetics, work‐function shifts, and the stability of reconstructed surface states under operating conditions. In this setting, charge equilibration or other physically grounded treatments of charge response are not merely “nice‐to‐have” features but can be critical for ensuring physically consistent potential‐dependent energetics, especially at electrified solid‐liquid interfaces. Although recent work [79, 80, 81] suggests viable routes to incorporate long‐range electrostatics, such capabilities are not yet standard in most pretrained universal MLIP models, and future improvements in this area will likely require coupling charge‐aware MLIPs with constant‐potential or grand‐canonical formulations rather than treating the atomistic systems as simple local inputs. Third, universal MLIPs offer opportunities for physical interpretability. For instance, attention weights in the attentive GNN potential could reveal which atoms or bonds dominate energetic contributions, potentially enabling automatic identification of the active site on a catalyst surface. Some equivariant MLIPs can be decomposed into body‐order terms, potentially clarifying many‐body effects in binding. Moreover, methods such as SHapley Additive exPlanations (SHAP) [114] or layer‐wise relevance propagation can further reveal which chemical environments drive specific predictions, thereby offering design insights into stability, reactivity, and surface reconstruction.

Lastly, validating MLIPs’ predictions against experimental data wherever possible. While experiments measure observables rather than PESs, predictions can still be tested against observables such as surface reconstructions (*via in situ* microscopy or spectroscopy), reaction rates, or diffusion coefficients. We also need to build trust further and reveal failures by closing the loop on experiments. Ultimately, universal MLIPs that consistently generate experimentally validated catalytic materials and mechanisms would be transformative for the field.

## 4 LLMs for Catalysis

### 4.1 The Rising LLMs Approach and Its Powerful Capability in Catalysis

**FIGURE 4:**
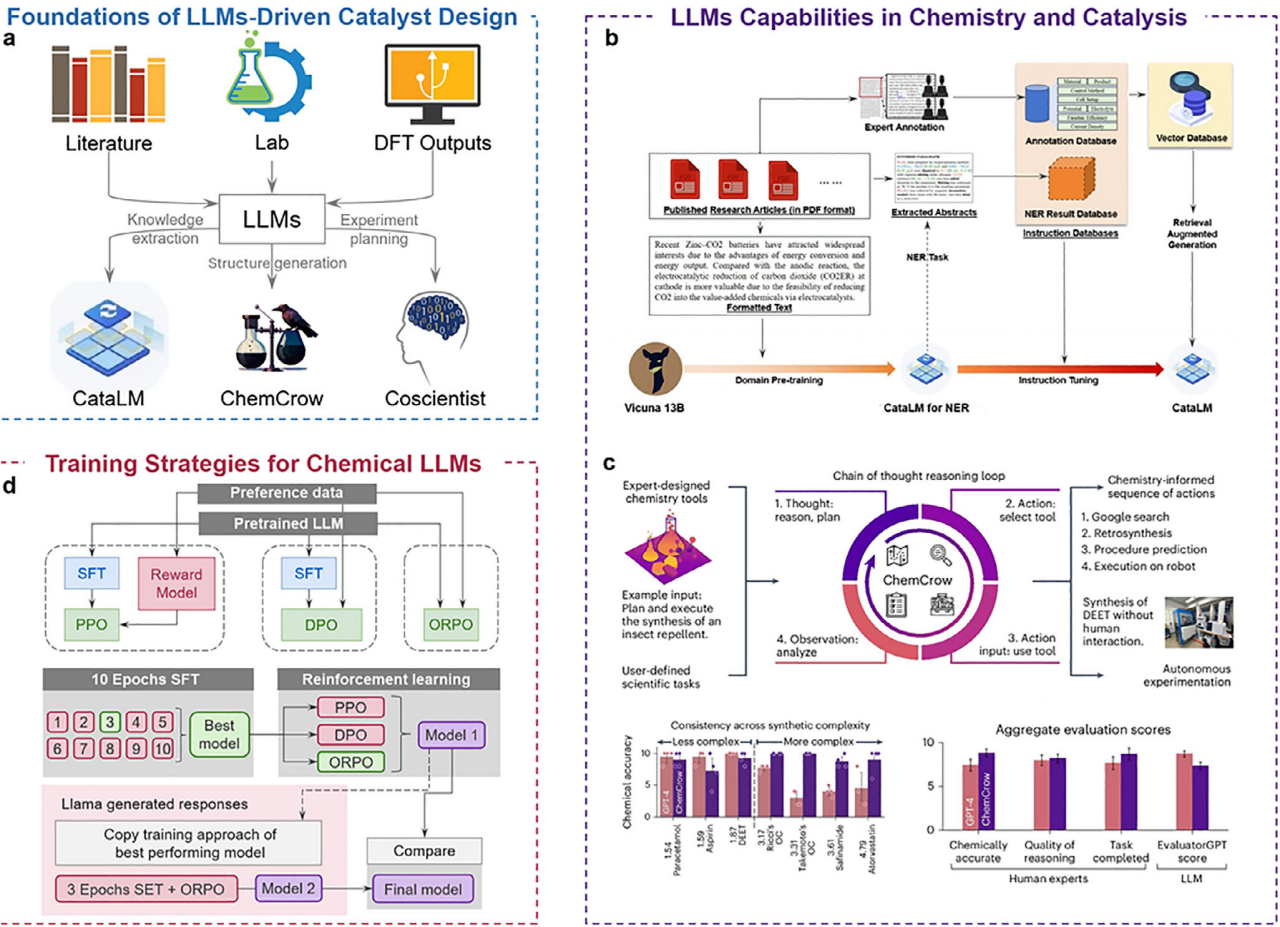
Catalyst design by LLMs. (a) Conceptual overview of catalyst design frameworks from traditional machine learning workflows to LLMs‐enabled discovery. (b) The training process of CataLM comprises the main training and the complete data preparation. Reproduced from Ref. [116]. Copyright 2025, Springer Nature. (c) Benchmark comparison of GPT‐4 and ChemCrow, summarizing per‐task preferences, chemical accuracy, aggregate human/Evaluator GPT scores, and qualitative strengths and weaknesses across synthesis, molecular design, and chemical‐logic tasks. Reproduced from Ref. [118]. Copyright 2024, Springer Nature. (d) Correlation analysis of model‐merging attributes (parent diversity, SFT, DPO/ORPO, base vs instruct) with merged performance, highlighting the substantial positive impact of SFT and the negative impact of excessive parent diversity. Adapted from Ref. [119]. (arXiv:2502.19347).

The rise of AI techniques, particularly transformer‐based LLMs, is opening new opportunities for catalysis research, especially in literature understanding, knowledge extraction, and workflow orchestration [8, 11, 12]. Importantly, most LLM‐for‐catalysis studies to date remain proof‐of‐concept demonstrations that rely on constrained tasks, curated toolchains, and substantial human oversight. Unlike coordinate‐dependent GNN/MLIP models, LLMs can operate on natural‐language descriptions and are best positioned as a flexible interface for retrieval, planning, and tool use in catalysis workflows (Figure 4a) [115]. By integrating knowledge derived from the literature, experimental records, and computational data, LLMs extend catalyst design beyond coordinate‐based representations by providing a text‐centered layer for integrating heterogeneous knowledge (literature, experimental records, and computational results) into actionable workflows. Within this framework, domain‐specialized LLMs have demonstrated strong capabilities in catalytic applications. One example is CataLM [116], an LLM explicitly trained for electrocatalytic materials, which demonstrates promise in literature‐grounded retrieval, candidate summarization, and interpretable assessments of catalyst performance through a two‐phase training protocol (domain pre‐training and fine‐tuning instruction) (Figure 4b). The CataLM model utilizes a domain‐specific catalytic literature dataset annotated by experts (containing more than 12,000 papers), which creates a specialized database of catalytic knowledge and improves the model's ability to understand and apply catalytic domain knowledge. In applying catalytic CO_2_ reduction for formic acid production, CataLM could accurately recommend copper nanoparticles and nitrogen‐doped graphene oxide (Cu/N‐GO) composites and elaborate on their synergistic mechanisms. This highlights the potential of domain‐specialized LLMs as expert‐facing assistants for knowledge synthesis and hypothesis generation in catalysis, rather than as standalone mechanistic predictors. Relatedly, LLMs have been used to support literature interpretation workflows in single‐atom catalysis toward advanced oxidation processes, further illustrating their utility in expert‐facing research assistance [117].

In automated scientific research, LLMs have also demonstrated powerful capabilities. For example, the Coscientist system, powered by GPT‐4, integrates multiple modules, including web search, document parsing, code execution, and experiment control, to build an intelligent research framework that functions as a “scientific assistant” [120, 121]. Its main module, “Planner,” understands research tasks through natural language processing, and calls sub‐modules such as “GOOGLE,” “DOCUMENTATION,” “PYTHON,” and “EXPERIMENT” to streamline the workflow from knowledge search to experiment execution through modular tool use and human‐defined constraints [122]. The system demonstrated closed‐loop optimization in scoped settings, with task definitions, safety constraints, and verification steps provided by human operators. The Coscientist demonstrates the potential of combining LLMs with automated experimental platforms to illustrate the potential of combining LLM planning with automated platforms for workflow automation and reproducibility; however, most current systems remain workflow‐level proof‐of‐concepts rather than fully autonomous scientific discovery engines.

Beyond prediction, LLMs can serve as an interpretation and interaction layer by organizing outputs into human‐auditable narratives and facilitating the use of explainability tools; however, interpretability does not automatically imply physically correct mechanistic reasoning [123]. The Interpretable Machine Learning (IML) framework helps extract physically meaningful relationships that models have learned [124, 125]. Esterhuizen et al. proposed “grey‐box ML” (*e.g*., SHAP) for post‐hoc variable attribution from “glass‐box ML” (*e.g*., symbolic regression and decision trees) that yield transparent structure‐property rules. Embedding these strategies in LLM‐driven workflows enhances the mechanistic credibility of catalytic predictions and supports hypothesis generation [126, 127].

LLMs are thus well‐positioned to accelerate data‐driven research. Data‐driven research leverages large‐scale, high‐dimensional datasets to learn statistical models that approximate structure‐performance relationships, which offer an alternative to the traditional research path that relies on intuition and trial‐and‐error experimentation. For example, in 2016, Raccuglia et al. pioneered the use of failed experiments (dark reactions) to train ML models to predict the crystallization success rate of inorganic‐organic hybrid materials, with a success rate of 89%, which is significantly better than traditional experimental strategies [128]. This work emphasized the importance of “negative samples” in model training and laid the foundation for subsequent data‐driven research, which is now being extended through LLMs. Thanks to their robust language understanding and knowledge‐integration capabilities, LLMs can extract key variables from unstructured sources such as the literature, laboratory notebooks, and sometimes even images. In materials chemistry, LLMs can integrate multi‐scale information, ranging from atomic‐level quantum‐chemical data (*e.g*., adsorption energies and reaction kinetic barriers) to macroscopic experimental results (*e.g*., current densities and product selectivities), thereby enriching the breadth and depth of models [12]. Building on this vision, Chattoraj et al. developed the LLM‐aided framework (AceWGS) for water‐gas shift catalysis, leveraging LLM‐based inverse modeling to identify high‐performance candidates [129]. Coupled with retrieval‐augmented generation (RAG), the integration of numerical and textual knowledge builds a robust foundation for cross‐disciplinary catalyst discovery.

However, while these advances point to an exciting new direction, they also raise some fundamental scientific questions. LLMs are primarily based on semantic associations learned from text, which differ significantly from the physical laws that govern atomic‐scale catalysis. As a result, the applicability of LLMs largely depends on how we address challenges related to accuracy, interpretability, generalization, diversity, experimental integration, and computational cost. Without careful examination, the field risks equating language fluency with mechanistic understanding, or corpus‐derived knowledge with validated scientific insights. To avoid overstatement, we distinguish demonstrated capabilities of LLMs in catalysis (literature understanding, knowledge extraction, workflow orchestration, and tool use) from aspirational roles (mechanism inference, quantitative barrier/rate prediction, and cross‐system extrapolation). In catalysis, hallucinations can manifest as plausible‐looking yet thermodynamically impossible or electrochemically inconsistent pathways, motivating rule‐based checks, physics‐grounded evaluators (DFT/MLIPs), and validation‐in‐the‐loop.

### 4.2 The Limitations and Obstacles of LLMs in Catalysis

#### 4.2.1 Accuracy Constraints From Corpus Bias and Incomplete Mechanistic Knowledge

Although LLMs show great potential for accelerating catalyst materials discovery, several key factors still constrain their practical application in catalysis. Generic models (*e.g*., ChatGPT) learn chemical concepts from massive Internet corpora but struggle to capture essential catalytic mechanisms, such as multi‐step electron/proton transfer, short‐lived intermediates, surface reconstruction, electric‐field effects, and solvation structures [12]. They tend to acquire broad but shallow domain knowledge and therefore lack relevance to tasks such as catalyst design, which require a specialized background and fine‐grained, mechanism‐resolved data. For example, Singh et al. noted that, despite impressive progress on natural language processing tasks, LLMs’ ability to generalize in scientific research remains limited [130]. Models trained only on general‐purpose text often fail on high‐precision materials‐science problems. They must be augmented with structured scientific knowledge, symbolic reasoning, and/or physics‐informed modeling to be trustworthy. Building on this, Jia et al. further emphasized that most current LLMs are trained mainly on text, making it difficult for them to interpret unstructured scientific content, such as microscopy images, spectra, or mechanistic schemes in electrocatalysis [12]. They highlighted the need for standardized, high‐quality datasets and for the fusion of multimodal information (*e.g*., text, images, and other data types) as key approaches to improving scientific understanding of a model.

Real catalytic processes are dynamic and multiphysics‐coupled (*e.g*., surface reconstruction, active site transferability, and evolving adsorption). Still, most LLMs rely on static inputs without trajectory, transition state, or electronic structure data, resulting in unreliable barrier and rate predictions. More broadly, LLMs mainly infer from semantic descriptions and lack rigorous, mechanism‐based theoretical derivation of transition states, competing pathways, and selectivity‐determining steps.

Therefore, the accuracy limitations of LLMs in catalysis do not stem solely from insufficient model size but from a fundamental discrepancy between statistical patterns in text and the underlying physical reality. Bridging this gap requires structured datasets, mechanism priors, and explicit integration of physical modeling (*e.g*., advanced microkinetic modeling) [131], rather than simply expanding the training corpus [132].

#### 4.2.2 Challenges in Interpretability and Lack of Physical Transparency

There is still a significant lack of interpretability in current LLMs applied to catalysis. Unlike traditional physicochemical models that explicitly describe activation barriers, bond rearrangements, or surface electronic structures, most LLMs operate as “black boxes,” making their mechanistic explanations difficult. This issue is especially evident in tasks involving electronic structure modulation, adsorption site differentiation, or pathway selectivity analysis. Esterhuizen et al. proposed that interpretability should be regarded as a key tool for scientific discovery, emphasizing the potential of grey‐box or glass‐box models to enhance models’ predictive power and physical plausibility [125]. This idea is essential for developing catalytic models that are mechanistically consistent and physically transparent. Liu et al. further demonstrated that domain‐knowledge‐infused prompt engineering can substantially improve structural expressiveness and semantic stability, partially alleviating the lack of explicit physical constraints [133]. Compared with generic prompting, structural prompts, reaction condition cues, and chemically specialized terminology significantly improve alignment between generated content and catalytic semantics. This ability is expected to deepen as structural databases and knowledge graphs mature.

In addition, Sprueill et al. explored interpretability using the Monte Carlo Thought Search with the ChemReasoner system, which combines an LLM with quantum‐chemical feedback mechanisms [134]. The methodology constructs a controlled knowledge space through heuristic search and reaction trajectory analysis, enabling a structured and modifiable reasoning process. In parallel, Singh et al. suggested that LLM‐based interpretability should shift from static visualizations of model predictions to more interactive, contextualized natural language explanations [127]. Such explanations, organized as chains or trees of reasoning that answer questions like “Why was this prediction made?” or “What are the alternative reasoning paths?”, can better support causal reasoning in scientific tasks and auditing in high‐stakes scientific applications.

Despite these advances, two key challenges remain. First, the generated content may exhibit severe hallucinations, i.e., explanations that are inconsistent with the actual prediction paths or lack a physical basis [135]. Second, the large and opaque architecture of current LLMs makes them incompatible with traditional interpretability tools such as saliency maps or attention visualization [136]. Future LLMs must move beyond purely physics‐ and chemistry‐free modeling by incorporating mechanistic priors such as reaction paths, charge redistribution, and active‐site structures, and by supporting verifiable, interactive explanations. This also makes coupling LLMs with physics‐based modeling tools a necessity.

#### 4.2.3 Obstacles to Generalizability Across Materials and Semantic Misalignment

The paradox of generalizability is a central challenge for applying LLMs in catalysis. Researchers aim to build broadly adaptable models across metals, alloys, X‐ides (X = O, N, C, *etc*.), and molecular catalysts; however, the cross‐system performance of current LLMs remains strongly dependent on corpus coverage, task‐structure consistency, and semantic alignment, making stable predictions difficult in complex reaction environments. For example, MatSciBERT, trained on ∼150,000 materials science publications, significantly outperforms general models such as BERT and SciBERT in domain‐specific NLP tasks [137]. However, because its training objective focuses on static literature mining, its ability to generalize to dynamic, multi‐step tasks such as reaction pathway evolution or interfacial structure analysis in catalysis remains unexplored. This reflects a broader challenge in deploying LLMs for catalysis: balancing domain specialization with cross‐task adaptability in complex reaction environments. Another typical example is ChemCrow, which achieves multitasking synergy, including property prediction, molecular design, and synthetic route recommendation, by integrating GPT‐4 with 18 chemistry tools, such as RDKit, the PubChem API, Thermo Tables, and ASKCOS (Figure 4c) [118]. Although the system demonstrates the potential to combine language models and toolchains for multistep tasks, its performance is highly dependent on the precision and configuration integrity of the tool calls. When dealing with catalytic systems with complex structures or unclear reaction paths, the system often suffers from execution failures and inference biases, exposing the limitations of generic architectures with insufficient transferability capabilities in multiple scenarios.

In contrast, many traditional MLIP models are less migratory, although primarily designed for specialized purposes and capable of achieving near‐*ab initio* accuracy in a particular catalytic system (*e.g*., a metal surface). Naik et al. point out that large‐scale general‐purpose models tend to be “generalized, but not sophisticated” and do not perform as well as optimized small models for complex or novel material systems, particularly in catalytic systems that are complex or ambiguous [138]. Optimized small models, particularly in path differentiation or boundary sample scenarios, exhibit a significant drop in accuracy. In the further exploration of multitask language models, Yang et al. pointed out that after fine‐tuning an LLM for a specific task, its ability to transfer and generalize to new tasks will be significantly affected. Especially in generative tasks, fine‐tuning often leads to a decline in the performance of the model in cross‐task and cross‐domain tests, indicating that its generalization ability is weakened.

Meanwhile, most LLM training corpus suffers from significant structural bias and semantic sparsity, especially in the subdomains of crystal defects, interface states, and solution reactions, and the problem of data scarcity restricts the effective generalization of the model in new scenarios. From the methodological perspective, relying on a single large model to cover all reaction systems is risky. As catalytic reaction types, electronic structure features, and mesoscopic environments become increasingly complex, it may be more feasible to construct a multi‐model synergistic system with unified structure encoding, mechanism‐label embedding, and task‐adaptation mechanisms [139].

#### 4.2.4 Impact of Missing Diversity Mechanisms on Complex Task Adaptation

Meanwhile, another critical but long‐neglected issue is the structured choice of model diversity, i.e., “whether one strong model should be developed that is transferable across domains, or whether multiple specialized models should be constructed to apply to different material systems”. Naik et al. proposed Diverse reasoning path Self‐Ensemble (DIV‐SE) and In‐call DIV‐SE (IDIV‐SE) strategies to systematically explore how diversity in reasoning paths can enhance the accuracy, robustness, and stability of LLMs in complex reasoning tasks [138]. They argue that relying on a single model or fixed decoding path limits the model's ability to capture diverse problem‐solving strategies, especially in high‐complexity settings. Instead, introducing structurally independent reasoning paths leads to better generalization and error tolerance. This perspective is particularly illuminating for catalytic reaction modeling, which often involves complex spatial structures and competing mechanisms. In this context, it may be beneficial to explore the integration paradigm, where multiple specialized models are trained for subtasks such as surface reaction prediction, interfacial structure modeling, and reaction path planning. These models can share information through a unified representation and coordination mechanism, thereby balancing accuracy and coverage. While this integration‐based paradigm extends beyond the original DIV‐SE framework, it echoes the core idea of employing multiple inference strategies to improve performance in complex environments.

On the other hand, Wang et al. pointed out from the perspectives of RAG and long context modeling that without semantic diversity, language models are prone to the problems of redundancy and narrow focus in information retrieval or content generation, resulting in the omission of key information or lack of factual integrity in the generated results [140]. They demonstrated a significant improvement in semantic diversity and result quality by introducing strategies such as Maximal Marginal Relevance (MMR) and Farthest Point Sampling (FPS) across several Q&A and summarization tasks. It is also crucial to incorporate information diversity mechanisms when constructing cue templates or context window structures for catalytic tasks, particularly for functions that depend heavily on contextual coverage, such as response path generation and interfacial state inference.

The current understanding of LLM diversity strategies is generally centered on linguistic decoding. However, in physically driven domains such as catalysis, the intrinsic mechanism space and response dimensions indicate that structured diversity warrants greater attention. Realizing unified scheduling across model architecture design, cue construction, knowledge retrieval, and a multi‐path thinking chain will be a key breakthrough in the future, promoting LLMs as a “mechanism‐aware system.”

#### 4.2.5 Limited Adaptability due to Model‐Experiment Integration Gaps

Although LLMs increasingly assist theoretical and computational chemistry, their integration into experimental workflows remains limited. In many experimentally driven settings, integrating LLM‐based toolchains into routine workflows still faces practical barriers, including resource and software maintenance, engineering effort for scripted workflows, and limited interface standardization across software and instruments [118]. This leaves much of the experimental work still dependent on researchers with computational backgrounds to implement it.

Even sophisticated platforms such as ChemCrow face practical barriers: complex installation, fragile toolchain dependencies, and unreliable function calling when dealing with intricate catalytic systems. For example, a standardized interface protocol (*e.g*., a REST API for interfacing with experimental equipment) must be developed if the generated reaction conditions are used in the instrument setup. Currently, this technology ecosystem is still fragmented, which hinders the construction of an efficient closed loop between experiment‐model‐automation systems. To promote the adaptability of LLMs in catalytic experimental processes, there is an urgent need to achieve breakthroughs in both technology integration and ease of use. This calls for low‐code tools, standardized interfaces, and real‐time validation loops to connect model outputs with experiments.

Therefore, the key to improving adaptability lies in optimizing the model structure and reasoning capabilities, and in reconfiguring the interaction logic and system architecture of LLMs to align with the experimental process. ChemCrow provides an attempt and highlights the key obstacles that must be overcome to get to a truly “experimental scenario‐compatible” language model. Suppose the feedback loop between “model‐human‐experiment” can be bridged in the future. In that case, LLMs are expected to become a truly usable, trustworthy, and controllable intelligent assistant for frontline researchers in experimental chemistry.

#### 4.2.6 Fine‐Tuning Challenges Under High Computational Cost Constraints

The high computational resource consumption has become a significant bottleneck limiting the practical use of LLMs in catalysis. Current mainstream models (*e.g*., GPT‐4 and LLaMA) contain billions of parameters and require substantial GPU memory, bandwidth, and arithmetic power for both training and inference. Domain fine‐tuning often relies on multi‐GPU clusters, resulting in long training cycles and high operational costs. Buehler et al. analyzed several approaches, including Continuous Pre‐Training (CPT), Supervised Fine‐Tuning (SFT), and Preferred Fine‐Tuning (PFT). They also analyzed the application of reinforcement learning, such as Direct Preference Optimization (DPO), Odds Ratio Preference Optimization (ORPO), and other strategies in models with different parameter sizes (Figure 4d) [141]. The results reveal that SFT exhibits the strongest positive correlation with performance improvement, whereas excessive parent‐model diversity negatively affects merged‐model performance, indicating diminishing returns from indiscriminate model aggregation. Reinforcement learning provides secondary gains but does not compensate for the adverse effects introduced by over‐diversification. These findings highlight a pronounced resource performance asymmetry in large‐model optimization.

One promising direction to reduce costs is Parameter‐Efficient Fine‐Tuning (PEFT). Among them, Low‐Rank Adaptation (LoRA), a representative approach, has demonstrated promising performance in tasks such as language generation, question answering, and coding [142]. It significantly reduces the explicit memory requirement by introducing a learnable low‐rank matrix rather than full‐volume parameter optimization, making it suitable for deployment in resource‐constrained environments. However, its performance on complex catalytic systems that require modeling of interfacial charge redistribution, multipath reactions, and transition states remains insufficiently validated. The recently proposed Directionally Optimized LoRA (DoRA) improves feature learning by decoupling magnitude and direction and outperforms standard LoRA on several benchmarks; however, its applicability to catalysis remains empirically untested [143]. As the model size and corpus volume expand, its performance gradually enters a “diminishing edge” phase. Buehler et al. point out that when the model is close to knowledge saturation, further performance enhancement often requires exponentially increasing resource inputs [138]. This asymmetry is particularly salient in resource‐constrained catalysis research environments. In addition, although the model's semantic generalization has improved, its ability to model structure‐driven mechanisms remains relatively limited, and its marginal contribution is small in tasks involving reaction pathway construction and selective modulation.

In conclusion, the asymmetry between computational resources and performance benefits will ultimately impact the sustainable development of LLMs in catalysis science. A promising direction is the use of medium‐scale models augmented by physics‐aware fine‐tuning, which incorporates structural priors (*e.g*., active sites, reaction coordinates, and charge distributions) and establishes bidirectional mappings between language semantics and catalytic mechanisms. Such models may offer a more sustainable balance between performance, cost, and mechanistic reliability.

## 5 Summary and Outlook

The integration of databases, MLIPs, and LLMs into catalysis has demonstrated remarkable potential for advancing research on catalytic materials. Compared with the traditional research paradigm, these AI database‐driven technologies offer unique advantages and capabilities for catalyst design and discovery, promising to accelerate catalyst discovery and development. In the era of large AI models, we comprehensively underscore how the AI‐empowered data science is revolutionizing the traditional catalysis paradigm and bridging the ontology‐concept‐computation‐experiment continuum, by the integration of MLIPs and LLMs. Using these advanced technologies, researchers can achieve highly efficient data acquisition, training, prediction, and even autonomous research in the field of catalysis. However, from potential to application, the current MLIPs and LLMs face many limitations in catalysis research.

Universal MLIPs are moving catalysis modeling beyond narrowly parameterized force fields toward broadly transferable, GNN‐based surrogates of the DFT PES that can handle many elements and environments relevant to heterogeneous catalysts, including solids, surfaces, and adsorbates. In this Mini‐Review, we use “universal MLIPs” to denote state‐of‐the‐art, multi‐element potentials trained on large, diverse datasets and refined for a catalytic design space, distinguishing them from true foundation models that are expected to adapt across downstream tasks and even levels of quantum theory. Achieving this versatility is increasingly driven by a data‐and‐training stack that combines large‐scale pre‐training with targeted fine‐tuning, often augmented by multi‐fidelity or delta learning to correct baseline‐DFT limitations, and by knowledge distillation to reduce cost for long MD runs. Because catalysis routinely generates out‐of‐distribution configurations, active learning with rigorous uncertainty quantification, robustness strategies (including adversarial or far‐from‐equilibrium sampling), and richer labels such as stresses, vibrational information, and charges are becoming central, alongside architectural physics priors such as equivariance and explicit treatments of long‐range interactions and electrostatics. These advances are enabling simulations that were previously impractical with DFT alone, including *operando*‐like surface reconstruction and active site evolution, free energy barriers and kinetics *via* enhanced sampling, and increasingly realistic solvent and electrified interfaces, even as field‐dependent and electrolyte‐general models remain a frontier. At the same time, the growth of leaderboards and benchmarks is clarifying that low static errors do not guarantee reliable catalytic trajectories, motivating stress tests that probe conservation, extrapolation, and long‐time stability. Looking forward, the key remaining challenges are building standardized, high‐fidelity surface and interface datasets across theory levels, integrating the right physics for stable long‐time dynamics without sacrificing accuracy, and improving interpretability and experimental validation so model predictions can be trusted, falsified, and translated into actionable catalyst design principles.

LLMs are promising and emerging tools for designing catalyst materials. With many recent efforts in the catalyst community, LLMs can enable text‐centered catalyst workflows that integrate literature, experiments, and computations. However, today's LLMs still face clear limits: patterns learned from text usually do not automatically match the physics of catalysis, so numerical accuracy can be unreliable, some explanations can be hard to trust, performance may drop when moving to new materials or tasks, and one single reasoning path often misses important alternatives. Practical issues also matter, including weak connections to real experimental workflows and the high cost of training and deployment, even with lighter fine‐tuning methods such as LoRA/DoRA. Looking ahead, steady progress will likely come from combining LLMs with physics‐based models, tightening the link between language outputs and mechanistic constraints, developing cheaper but reliable tuning methods, and building standard closed‐loop systems where models propose ideas, experiments test them, and the results are used to improve the next round of predictions. High‐quality corpora and well‐designed reasoning libraries will also be essential to support reliable learning and more consistent scientific inference.

Looking ahead, we propose the AI × Database closed‐loop platform that connects databases, MLIPs/LLMs, and automated experiments to iteratively improve catalyst discovery: **i) Data Infrastructure**: Integrating experimental and computational data into dedicated materials databases to serve as a unified foundation; **ii) AI + Theory**: Applying universal MLIPs, multiscale modeling, multi‐modal LLMs, and advanced algorithms to enhance predictive accuracy and mechanistic understanding; **iii) Closed‐Loop Innovation**: Connecting digital platforms, autonomous synthesis, and high‐throughput experimentation into a self‐iterating “prediction—validation—feedback” cycle, bridging fundamental research and industrial application.

Additionally, by consolidating and interlinking datasets from diverse materials subfields, we propose constructing a Digital Materials Ecosystem [144] that covers a broader range of material domains. By integrating MLIPs, LLMs, and machine learning algorithms, this system can bridge the catalysis database and other material databases, significantly broadening the scope of catalyst predictions and facilitating their adaptation to previously unexplored domains. This ecosystem moves beyond traditional exploration within a single field, paving the way for innovative advances in catalysis science.

We believe that AI‐empowered catalysis research will be propelled by integrated universal MLIPs, multimodal LLMs, and automation‐driven data‐mining systems, opening a new era of transformative breakthroughs in catalysis research. As AI‐enabled universal MLIPs and LLMs become increasingly accessible and sophisticated, they may eventually enable real‐time, AI‐driven automated design, fostering an era in which catalyst discovery and application reach unprecedented heights.

## Conflicts of Interest

The authors declare no conflicts of interest.

## Data Availability

Data sharing is not applicable to this article as no new data were created or analyzed in this study.
